# 

**Published:** 1893-05

**Authors:** 


					﻿Vol XIII.
MAY, 1893.
NO. 5.V
THE
JIOMEOPATHIC pHY^lClA]3,
A Monthly Journal of Homoeopathic Materia Medica
and Clinical Medicine.
"He is a freeman whom truth makes free, and all are slaves beside."
"Seek the Truth: come whence it may, cost uhat it will."
EDITED AND PUBLISHED BY
WALTER M. JAMES,' M. D.
PHILADELPHIA :
No. 1125 SPRUCE STREET.
LONDON:
ALFRED HEATH & CO., Sole Agents fob Gekat Britain,
114 Ebury Street, Eaton Square.
SEgT Yearly Ssebsersption, $2.50, invariably in advnee. Single numbers, 25 ets.
Enterea at the Philadelphia Poet-Oftce a* Second Claaa Mai! Matter.
				

